# Efficacy and safety of proxalutamide (GT0918) in severe or critically ill patients with COVID-19: study protocol for a prospective, open-label, single-arm, single-center exploratory trial

**DOI:** 10.1186/s40360-023-00678-7

**Published:** 2023-06-15

**Authors:** Dawei Yang, Minjie Ju, Hao Wang, Yichen Jia, Xiaodan Wang, Hao Fang, Jia Fan

**Affiliations:** 1 Department of Pulmonary and Critical Care Medicine, Zhongshan Hospital Fudan University, Shanghai, China; 2 Department of Pulmonary and Critical Care Medicine, Zhongshan Hospital (Xiamen), Fudan University, Xiamen, China; 3 Shanghai Engineer & Technology Research Center of Internet of Things for Respiratory Medicine, Shanghai, China; 4 Department of Critical Care Medicine, Zhongshan Hospital Fudan University, Shanghai, China; 5 Department of Thoracic Surgery, Zhongshan Hospital Fudan University, Shanghai, China; 6 Department of Urology, Zhongshan Hospital Fudan University, Shanghai, China; 7 Department of Anesthesiology, Zhongshan Hospital Fudan University, Shanghai, China; 8 Department of Anesthesiology, Minhang Hospital, Fudan University, Shanghai, China; 9 Department of Liver Surgery and Transplantation, Liver Cancer Institute, Zhongshan Hospital, Fudan University, Shanghai, China; 10 Key Laboratory of Carcinogenesis and Cancer Invasion of Ministry of Education，Fudan University, Shanghai, China; 11 Key Laboratory of Medical Epigenetics and Metabolism, Institutes of Biomedical Sciences, Fudan University, Shanghai, China

**Keywords:** COVID-19, Critical ill COVID-19, Proxalutamide, Severe COVID-19

## Abstract

**Background:**

The rapid worldwide spread of COVID-19 has caused a global health challenge with high mortality of severe or critically ill patients with COVID-19. To date, there is no specific efficient therapeutics for severe or critically ill patients with COVID-19. It has been reported that androgen is related to severe acute respiratory syndrome coronavirus 2 (SARS-CoV-2) infection. Proxalutamide as an androgen receptor antagonist has shown potential treatment effects on COVID-19 patients. Thus, this trial is designed to investigate the efficacy and safety of proxalutamide in severe or critically ill patients with COVID-19.

**Methods:**

This single-arm, open-label, single-center prospective exploratory trial is planned to recruit 64 severe or critically ill patients with COVID-19 in China. Recruitment started on 16 May 2022 and is foreseen to end on 16 May 2023. Patients will be followed-up until 60 days or death, whichever comes first. The primary outcome is the 30-day all-cause mortality. Secondary endpoints included 60-day all-cause mortality, rate of clinical deterioration within 30 days after administration, time to sustain clinical recovery (determined using an 8-point ordinal scale), mean change in the Acute Physiology and Chronic Health Evaluation II scores, change in oxygenation index, changes in chest CT scan, percentage of patients confirmed negative for SARS-CoV-2 by nasopharyngeal swab, change in Ct values of SARS-CoV-2 and safety. Visits will be performed on days 1 (baseline), 15 or 30, 22, and 60.

**Discussion:**

The trial is the first to investigate the efficacy and safety of proxalutamide in severe or critically ill patients with COVID-19. The findings of this study might lead to the development of better treatment for COVID-19 and provide convincing evidence regarding the efficacy and safety of proxalutamide.

**Trial registration:**

This study was registered on 18 June 2022 at the Chinese Clinical Trial Registry (ChiCTR2200061250).

## Background

In 2019, a novel coronavirus called severe acute respiratory syndrome coronavirus 2 (SARS-CoV-2) caused the COVID-19 pandemic outbreak, which turned into a massive global health challenge. According to the weekly epidemiological updates of the World Health Organization till 24 May 2023, approximately 766 million cases were confirmed worldwide, resulting in about 6.9 million deaths [1]. The clinical features of COVID-19 are diverse and range from asymptomatic to death, and severe and critically ill patients represented 14% and 5%, respectively [2]. The mortality for critical cases reached 60.5% according to a recent report [3]. Currently, in patients with moderate to severe COVID-19, the major available therapeutics are drug treatments, including antiviral agents, inflammation inhibitors/antirheumatic drugs, hyperimmune immunoglobulins, and so on. However, most studies of existing drug therapy have limited therapeutic effects and lack compelling evidence [4–6]. A systematic review of the antiviral drug remdesivir for patients with severe COVID-19 found that given the current evidence, the efficacy data of remdesivir in severe COVID-19 is insufficient [7]. Therefore, it is urgently necessary to identify an effective drug to combat the disease considering the grim situation.

Infections by the SARS-CoV-2 are dependent on transmembrane protease serine 2 (TMPRSS2) and host proteins angiotensin-converting enzyme 2 (ACE2) receptor [8]. It has been demonstrated androgen are the only known transcription promoters for the TMPRSS2 gene [9]. Moreover, it has also been shown that ACE2 activity reduction is associated with androgen decrease [8]. Several studies showed that androgen-mediated androgenetic alopecia (AGA) was associated with more severe COVID-19 diseases, which has been demonstrated in several studies in hospitalized men patients [10–12]. Thus, androgen plays a crucial role in SARS-CoV-2 infection and the androgen receptor (AR) blockers would likely improve the treatment effect of COVID-19.

Proxalutamide is a potent second-generation nonsteroidal androgen receptor antagonist, which has a better therapeutic effect than other antiandrogen compounds (bicalutamide and enzalutamide) [13, 14]. Previous studies also reported the use of proxalutamide in COVID-19. In a double-blinded, placebo-controlled, randomized clinical trial, females treated with proxalutamide have reduced hospitalization rates [15]. In another study in patients with mild to moderate COVID-19, proxalutamide significantly accelerates viral clearance and reduces the time to clinical remission [16]. However, most existing studies on proxalutamide in COVID-19 have focused on patients with mild to moderate [15–17]. There are no studies on the efficacy and safety of proxalutamide in the treatment of severe or critically ill patients with COVID-19. Therefore, a single-arm, open-label, single-center prospective exploratory trial is designed to investigate the efficacy and safety of proxalutamide in the treatment of severe or critically ill patients with COVID-19 in China.

## Methods

### Study design

This is a single-arm, open-label, single-center prospective exploratory trial to assess the efficacy and safety of proxalutamide in severe or critically ill COVID-19 subjects. Recruitment started on 16 May 2022 and is foreseen to end on 16 May 2023. This trial is designed according to the Standard Protocol Items: Recommendations for Interventional Trials (SPIRIT) 2013 Statement [18]. In addition, this trial is registered in the China Clinical Trial Registry (ChiCTR2200061250).

### Trial setting

The trial is being conducted in the Gerontology Center of Zhongshan Hospital affiliated to Fudan University. The center is a 2000-bed tertiary general hospital located in Shanghai, China, which is a temporarily designated hospital for the treatment of patients with COVID-19 during the COVID-19 pandemic. The hospital is responsible for patients with moderate COVID-19 as well as patients with underlying diseases and mental illness and sets up COVID-19 patients with mental illness wards and intensive care unit (ICU) wards to admit critically ill patients.

### Participant recruitment

Participants will be recruited by physicians in the Gerontology Center of Zhongshan Hospital affiliated to Fudan University. Potentially eligible patients will be identified by trained physicians using an automated search and will receive a preliminary participant information sheet about the trial. Participants will be given a full explanation and time to consider before entering the trial, and if they agree, a written consent form will be signed. In addition, if the patient lacks the capacity to provide informed consent, a legal representative will be asked to provide consent. On the consent form, participants will be asked for their permission to share relevant data whether they withdraw from the trial or not. All written informed consent forms will be taken by a research nurse or a trained medical professional.

### Participant eligibility

Inclusion criteria are as follows:

- (1) Participants aged ≥ 18 years;
- (2) Participants with laboratory-confirmed SARS-COV-2 infection by reverse transcription PCR (RT-PCR);
- (3) Participants who are diagnosed with severe or critically ill COVID-19 according to the diagnosis and treatment protocol for COVID-19 patients (Trial Version 9) [19]. Table 1 lists the diagnostic criterion for severe or critically ill COVID-19 patients;
- (4) Contraception is maintained during treatment and for 90 days after the last dose of the study drug;
- (5) Participants (or legally authorized representatives) who sign the informed consent form before starting any procedure.

**Table 1:** The diagnostic criterion for severe and critically ill COVID-19

Severe COVID-19 is defined as (no matter how long the condition lasts, at least one of the following conditions):
• Shortness of breath and respiratory rate ≥ 30 breaths/minute • Clinical symptoms progressively worsen, and lung radiographic infiltrates by imaging (eg, chest x-ray, CT scan.) show > 50% lung infiltrates within 24–48 h • PaO_2_) / (FiO_2_) ≤ 300 mmHg (1 mmHg = 0.133 kPa) • Oxygen saturation ≤ 93% in resting state
Critically ill COVID-19 is defined as (at least one of the following conditions):
• Respiratory failure (defined as the need to receive at least one of the following treatments according to local medical resources/conditions: endotracheal intubation and mechanical ventilation, high-flow nasal catheter oxygen supply (heating, humidification, enhanced nasal catheter oxygen supply, flow rate > 20 L/minute, oxygen supply ratio ≥ 0.5), positive pressure ventilation, ECMO, or clinical diagnosis of respiratory failure (defined as none of the above treatments cannot provide due to limited medical resources) • Shock (defined as systolic blood pressure < 90 mm Hg, diastolic blood pressure < 60 mm Hg, or the need for vasopressors) • Multiple organ dysfunction/failure

The diagnostic criterion for severe or critically ill COVID-19 patients can be adjusted according to the clinical diagnosis and treatment guidelines for COVID-19

Exclusion criteria are as follows:

- (1) Participants who have participated in other clinical studies of proxalutamide;
- (2) Anine aminotransferase (ALT)/aspartate aminotransferase (AST) > 5 times the upper limit of normal (ULN);
- (3) Serum total bilirubin > 1.5 × ULN;
- (4) Estimated glomerular filtration rate (eGFR) < 30 mL/min (including patients undergoing hemodialysis or hemofiltration);
- (5) Participants with suspected serious active bacterial, fungal, viral, or other infections (except infections of COVID-19), which may pose a risk when taking the study drug according to the opinion of investigators;
- (6) Participants with a known history of the human immunodeficiency virus (HIV), active hepatitis B, or active hepatitis C;
- (7) Pregnant or lactating women;
- (8) Participants with terminal underlying diseases who are not suitable for the study as assessed by the doctor;
- (9) Participants with a planned pregnancy within 90 days.

### Intervention

Eligible patients will receive Proxalutamide (300 mg, Kintor Pharmaceuticals Ltd., Suzhou, China) plus standard of care. Proxalutamide will be administered orally once daily at the same time each day (± 2 h according to medication scheduling). The duration of the study treatment will last for 7 days and can be extended to 14 days at the discretion of the investigator.

Any dose that is missed and delayed may be administered as soon as possible on the same calendar day. When it is almost time for the next scheduled dose (within 4 h), we recommend skipping the missed dose and taking the next dose instead. The missed dose will not be made up if it is not given on the same calendar day.

Use of antipyretic medications (eg, paracetamol) or other adjunctive medications (eg, vitamins) is allowed before enrollment. Use of the following drugs is allowed during the trial period: treatment drugs against adverse events as well as underlying conditions (used stably for at least 3 months); oral contraceptive; convalescent plasma from recovered COVID-19 patients; anti–SARS-CoV-2 monoclonal antibodies; corticosteroids. Moreover, participants are advised to avoid the consumption of grapefruit, carambola, bigarade, or their juice, which belongs to CYP3A4 inhibitors and can increase exposure to proxalutamide. Allowable drugs for severe or critically ill patients with COVID-19 are administered based on investigators’ clinical judgment and clinical practice guidelines [20].

Discontinuance criteria of treatment are as follows: (1) If participants are tested negative for SARS-CoV-2 by RT-PCR assay or Ct values > 35, and repeated testing is still negative or Ct values > 35 after 24 h, the treatment will be discontinued by the investigator; (2) If grade 3–4 drug-related adverse events (AEs) occur during treatment, proxalutamide will be permanently discontinued.

It is important for the patient to fully understand the importance of conducting the trial, medicine administration on time, and follow-up on schedule during the trial for enhancing patient adherence. The subjects will be required to take the medicine according to the regulations, fill in the patient diary card on time, and attend the follow-up as required.

### Study endpoints

The primary endpoint of this study is 30-day all-cause mortality. Secondary outcomes include the following:

- (1) 60-day all-cause mortality;
- (2) The proportion of clinical deterioration, which is defined as the need within 30 days after administration for admission to the intensive care unit or invasive mechanical ventilation/ extracorporeal membrane oxygenation (ECMO), or all-cause mortality;
- (3) Mean change in clinical status using the 8-point-ordinal-scale from day 1 (baseline) to day 15 and day 22 (optional);
- (4) Time to sustain clinical recovery (determined using an 8-point ordinal scale);
- (5) Percentage of subjects reaching sustained clinical recovery (determined using an 8-point ordinal scale) on days 5, 8, 15, and 22 (optional);
- (6) Mean change in the Acute Physiology and Chronic Health Evaluation II (APACHE-II) scores from day 1 (baseline) to days 15 and 22 (optional);
- (7) Changes in oxygenation index measured by blood gas analysis or blood oxygen saturation from day 1 (baseline) to days 3, 5, 8, 15, and 30 (optional);
- (8) Changes in chest CT scan (optional) from baseline to day 8 or 15;
- (9) Percentage of subjects confirmed negative for SARS-CoV-2 RT-PCR by nasopharyngeal (NP) swab on days 3, 5, 8, 15, and 30 (optional);
- (10) Change in Ct values of SARS-CoV-2 RT-PCR on days 3, 5, 8, 15, and 30 (optional);
- (11) Safety assessments (eg, AEs, serious AEs, and abnormal laboratory data) from the time of SARS-CoV-2 RT-PCR turned negative to day 30.

### Follow-up and data collection

Patients will be followed-up until 60 days or death, whichever comes first. In this trial, data collection will occur on days 1, 3, 5, 8, 15, 22, 30, and 60. The schedule of data collection is shown in Table 2. Efficacy assessment includes all-cause mortality, clinical status (8-point ordinal Scale), APACHE-II scores, oxygenation index, changes in chest CT scan, and the clinical performance of nasopharyngeal severe acute respiratory syndrome coronavirus 2 reverse transcription polymerase chain reaction (NP SARS-CoV-2 RT-PCR) testing.

**Table 2:** Schedule of patient assessments

Time Point	Screening	Treatment Period		Safety Follow-up				
		Baseline (Day 1)	Day 2-Day 7 (up to Day 14)	Day 15 ± 2	Day 22 ± 2		Day 30 ± 3	Day 60 ± 3
Informed consent	●							
Verify eligibility criteria	●							
Demographics	●	●						
Medical History	●	●						
Targeted physical exam	●	●						
Review SARS-CoV-2 results	●	●						
Review X-ray or CT if applicable	●	●						
Vaccination status	●	●						
Vital signs including SpO_2_	●	●	Once a day till discharge	●			●^a^	
Targeted Physical Examination		●	●	●				
Clinical data collection		●	●	●	●^a^			
AEs evaluation		●	Once a day till discharge	●	●		●	●
Concomitant medication review		●	Once a day till discharge	●	●		●	
X-ray or CT			●^a^	●^a^				
12-ECG	The decision is made by professional investigators							
Hematology, urine routine, and coagulation action test	●	●	Day 3, 5, 8 (all ± 1 day)	●		●		
Pregnancy test for females of childbearing potential	●					●		
Nasopharyngeal swab		●	Day 3, 5, 8 (all ± 1 day)	●		●^a^		

*AEs* adverse events, *CT* computed tomography, *ECG* electrocardiography, *PI* principal investigator, *SARS-CoV-2* severe acute respiratory syndrome coronavirus 2

^a^ optional; ● mandatory

The safety assessment includes general vital signs, physical examination, 12-lead electrocardiography (ECG), laboratory examination (routine blood test, blood biochemical, routine urine test, and biomarker analysis) chest radiography, CT scans, and AEs. The physical examination includes assessments of the eyes, ears, nose, mouth, throat, cardiovascular, respiratory, gastrointestinal, genitourinary, hematologic/lymphatic, endocrine, and neurological.

The 8-point ordinal scale is an assessment of the clinical status at the first assessment of a given study day. The scale is defined as follows: (1) Death; (2) Hospitalized, on invasive mechanical ventilation or ECMO; (3) Hospitalized, on non-invasive ventilation or high flow oxygen devices; (4) Hospitalized, requiring supplemental oxygen; (5) Hospitalized, not requiring supplemental oxygen- requiring ongoing medical care (COVID-19 related or otherwise); (6) Hospitalized, not requiring supplemental oxygen, and no longer requires ongoing medical care; (7) Not hospitalized, limitation on activities; (8) Not hospitalized, no limitations on activities [21].

APACHE II score is a severity score and mortality estimation tool developed from a large sample of ICU patients, which consists of an acute physiology score (APS), the age of the patient, and chronic health points. And APACHE II score is made of 12 physiological variables and 2 disease-related variables. The maximum APACHE-II score is 71, though it is rare for a patient to score higher than 55. When APACHE II scores are combined with an accurate description of the disease, they can prognostically stratify acutely ill patients and assist investigators in comparing the success of new or differing forms of therapy [22, 23].

Nasopharyngeal swabs will be performed following a standardized procedure in this trial [24]. In addition, the result of NP SARS-CoV-2 RT-PCR will be adopted, if patients have already tested for NP SARS-CoV-2 RT-PCR no more than 48 h before the first drug administration. The viral clearance of COVID-19 in the treatment period defines as the results of both tests (the first test and retested 24 h later) being negative SARS-CoV-2 RT-PCR test or Ct values > 35.

### Plans to promote participant retention

If patients are discharged from the hospital and are unable to return for follow-up as scheduled due to being isolated in the COVID-19 epidemic, a visit on days 15 or 30 should be performed as quickly as possible after ending the isolation period. A face-to-face visit is preferred on day 22, nevertheless, quarantine and other factors should also be considered for return visit patients and a remote visit may be optional. The telephone visit may be conducted on day 60.

### Data management

All the data obtained for analysis in the clinical study described in this protocol will be recorded in the paper case report format (CRF). All paper CRFs are sent to Contract Research Organization (CRO) and then entered into the electronic data capture (EDC) system. Any fill-in, change, or correction to a CRF will be decided by the investigator or a person designated by the investigator. Investigators have demonstrated the accuracy of all recorded data. The Data in this study will be entered directly into CRFs and not be used as original medical records unless otherwise stated. The inspector will check the CRF to make sure all data recorded in the CRF is consistent with the original material. The data will be validated by the data management plan. To ensure the accuracy of the data, the quality control process will run through every stage of data processing.

### Sample size calculation

The primary endpoint of this study is the 30-day all-cause mortality. Based on a previous study, the all-cause mortality rate of hospitalized patients infected with COVID-19 is approximately 7.3% to 14.1% [25, 26]. Assuming that the proportion of all-cause mortality is 12% in severe or critically ill patients infected with COVID-19 under standard therapy, it is expected that the proportion of all-cause mortality can be reduced to 2% after treatment with proxalutamide. The probability of a type I error is set at 0.05 (*p* = 0. 05) with a power of 80%, and 58 patients will be required to be enrolled based on the exact probability method. It is expected that the drop-out rate of enrolled subjects will be ≤ 10%. Thus, this study is planned to recruit 64 severe or critically ill patients with COVID-19.

### Statistical analysis

All efficacy analysis results are analyzed in the full analysis set (FAS) and per-protocol set (PPS). The FAS is defined as all randomized patients who received at least one dose of the study drug. The PPS is defined as all patients who completed the treatment without serious violation of the protocol. The safety analysis will be performed based on the FAS and no imputation will be made for missing safety data. Categorical data will be expressed as numbers and percentages, and quantitative data will be expressed as means ± standard deviation (SD), median, interquartile range (IQR) values, or minimum and maximum. The primary outcome will be an analysis of the percentage of 30-day all-cause mortality. The analysis of efficacy in the secondary endpoint measures will be based on their variable attributes in an appropriate way. For binary variables, the exact probability method will be used to calculate the probability of the subject with clinical deterioration. For continuous variables, the value of actual measured values and the chances from the baseline will be summarized. For rank variables such as changes in the score of the 8-point order scale, the paired Signed Rank Test method will be used. For time-to-event variables, the Kaplan–Meier method will be used to estimate the median and Kaplan–Meier graphs will be provided. All statistical analyses will be performed using Statistical Analysis System 9.4 (SAS Institute, Inc, Gary, NC), and statistical significance was set at *p* < 0.05.

### Safety analysis

Adverse events are coded using the medical dictionary for regulatory activities (MedDRA) [27]. In this trial, statistical analysis is mainly performed for treatment-emergent adverse events (TEAEs), and pretreatment adverse events will be listed in a list. The number and proportion of all adverse events, drug-related adverse events, serious adverse events, severe adverse events, and adverse events leading to discontinuation of the drug will be calculated. Adverse events will be classified and statistically analyzed at two levels: system organ class (SOC) and preferred term (PT).

### Confidentiality

All communications, reports, and subject samples will be identified by site number, code number, and initials to maintain subject confidentiality. All records will be kept confidential to the extent permitted by law. If a waiver or authorization separate from the statement in the informed consent is required for permitting access to the medical records of a subject, the investigator will obtain such authorization before enrolling the subject in the study. The investigator should keep a separate log of subjects, codes, names, and addresses. Documents that identify the subject by name (eg, the informed consent forms) will be kept in strict confidence.

The sponsor and its business associates agree to keep all subject information confidential. Data resulting from analyses will be entered into a database that is not accessible to the public. Subject data will be identified only by the subject screen number, randomization number, and initials, and not by any other annotation or identifying information. The sponsor and its business associates will take every possible step to reduce the risk of releasing information to the public that would enable subjects to be personally identified.

### Interim analyses

There are no planned interim analyses.

### Oversight and monitoring

In this trial, the investigator or whose representative will conduct regular monitoring visits to evaluate whether the study is implemented in compliance with the study protocol according to good clinical practice (GCP) and whether the data are appropriately corrected. To make sure validation of the data, the investigator and institutions will allow their representatives, as well as relevant regulatory authorities, direct access to the original documents for data validation. The trial center requires review by an independent research ethics committee or institutional review boards and a relevant regulatory agency.

### Dissemination plans

The principal investigators will be responsible for the publication of the data and are committed to publishing and disseminating it promptly without excessive restriction. Findings will be published in peer-reviewed journals and disseminated through various channels including social media and at national and international meetings. The proposed publication should not include any sponsor information or any personal data of any subject (eg, name or initials) except the study results.

## Discussion

COVID-19 is a new and poorly understood disease that posed a huge challenge to global public health. Most patients with COVID-19 have mild symptoms, but some patients rapidly develop severe and critical illnesses, and the mortality of critically ill patients with COVID-19 is high [28]. Previous reports revealed that androgen-mediated expression of ACE-2 and TMPRSS2 related to the degree of severity and mortality of COVID-19 disease [29–31]. Proxalutamide as a novel potent AR antagonist has been shown to downregulate the expression of ACE2 and TMPRSS2 in lung cancer cells, and also accelerate viral clearance in mild to moderate patients with COVID-19 [32]. But the efficacy of proxalutamide treatment for severe and critically ill COVID-19 patients is unclear. Therefore, a single-arm, open-label, prospective exploratory trial is designed to investigate the efficacy and safety of proxalutamide in the treatment of severe and critically ill patients with COVID-19 for the first time.

This trial has several strengths. First of all, this is the first prospective study of proxalutamide in the treatment of severe or critically ill patients with COVID-19 in China. Currently several clinical trials of proxalutamide in COVID-19 are focused primarily on mild to moderate patients, all patients without the severity of illness, or mechanism of action for proxalutaminde treatment [16, 33, 34]. Our study will provide sufficiently strong evidence to guide the treatment of severe or critically ill patients with COVID-19. Secondly, there are no specific therapeutic agents or vaccines for COVID-19 available. This trial study on androgen receptor antagonists which is a novel therapeutic target in COVID-19, inhibiting SARS-CoV-2 infection by regulating ACE2 and TMPRSS2. It may provide a basis and reference for other androgen receptor drugs with similar mechanisms in COVID-19. Moreover, this study allows patients to use convalescent plasma from recovered COVID-19 patients, SARS-CoV-2 monoclonal antibody therapy, and corticosteroid during the treatment period. Under these conditions can represent the real world of the treatment of COVID-19 as accurately as possible.

Despite the above strengths, there are still some limitations in the present study. Firstly, our sample size was small and the trial was conducted at one center. However, as the COVID-19 pandemic is a sudden and temporary event, the center for accepting and treating severe or critically ill patients with COVID-19 is limited and temporally, meanwhile, the number of recruited severe or critically ill patients at clinical centers may be low. Secondly, this trial did not include a control group, therefore, reducing the generalizability of our results. Because there is no approved effective conventional treatment for patients with severe or critical COVID-19. Given the ethical considerations of protecting the maximum benefits of participants and the objectives (rapid discovery of proxalutamide efficacy) of this investigator-initiated clinical trial, no control group was recruited for this study. Randomized controlled trials will continue to be conducted in the future to assess the effect of proxalutamide. Thirdly, this is an open-label trial, considering severe or critically ill patients with COVID-19 may deal with invasive treatment delivered in the ICU (including ECMO), which is undoubtedly more difficult or impossible to conduct a blinded trial. But blind will be maintained for all other members of the clinical and research team, such as statistical staff, to minimize bias.

In summary, the present trial is the first to investigate the efficacy and safety of proxalutamide in severe or critically ill patients with COVID-19. The findings of this study might provide convincing evidence regarding the efficacy and safety of proxalutamide on severe or critically ill patients with COVID-19.

### Trial status

Participant recruitment started on 16 May 2022, with 64 participants recruited by 16 May 2023.

## Data Availability

The datasets during the current study are available from the corresponding author on reasonable request after the main results have been published, as long as it corresponds with the local rules and regulations for data sharing.

## Abbreviations

AR: Androgen receptor
ACE2: Angiotensin-converting enzyme 2
AEs: Adverse events
COVID-19: The 2019 coronavirus
DMP: Data Management Plan
EGG: Electrocardiography
ECMO: Extracorporeal membrane oxygenation
eGFR: Estimated glomerular filtration rate
ICU: Intensive care unit
IIT: Investigator-initiated trial
PPS: Per-protocol set
PT: Preferred Term
TMPRSS2: Transmembrane protease serine 2
TEAE: Treatment-emergent adverse event
TESAE: Treatment-emergent serious adverse event
ULN: The upper limit of normal
SOC: System organ class
